# Tumors of the Aorta as a Rare Cause of Ischemic Stroke in a Young Woman

**DOI:** 10.1016/j.jaccas.2025.103329

**Published:** 2025-04-09

**Authors:** Piera Ciaramella, Sabrina Anticoli, Domenica Crupi, Amedeo Pergolini, Marco Russo, Maria Cristina Macciomei, Norman Veccia, Antonio Lio, Federico Ranocchi

**Affiliations:** a Department of Cardiac Surgery and Heart Transplantation, San Camillo Forlanini Hospital, Rome, Italy; b Department of Cardiology, La Sapienza University of Rome, Umberto I Hospital, Rome, Italy; c Department of Head, Neck and Neuroscience, San Camillo Forlanini Hospital, Rome, Italy; d Department of Pathological Anatomy and Histology, San Camillo Forlanini Hospital, Rome, Italy

**Keywords:** aorta, cancer, stroke, stroke volume

## Abstract

Primary malignant tumors of the aorta represent a group of sarcomas arising from the aortic wall. Onset typically occurs in the fifth and sixth decades of life, and the prognosis is poor. Herein, we present the case of a 33-year-old patient with an acute neurological syndrome of uncertain origin. She underwent thrombolysis and mechanical thrombectomy of the left M1 cerebral artery and was discharged home with antiplatelet and anticoagulation therapy. After 30 days, follow-up imaging showed thrombotic-like formations in the ascending aorta, so she underwent urgent cardiac surgery. The histological and immunohistochemical studies showed an unexpected diagnosis of intimal sarcoma. A multidisciplinary approach was mandatory to define the timing of diagnosis and treatment.

## Graphical Abstract

**Figure:**
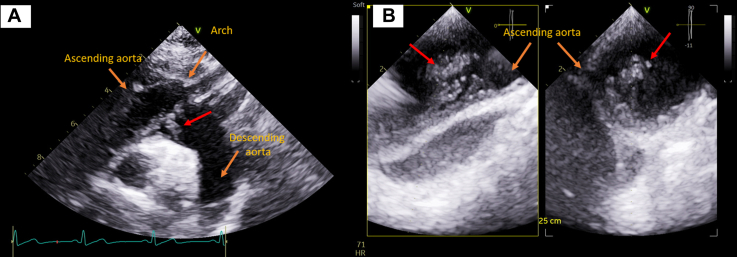

## History of Presentation

A 33-year-old female patient arrived at the emergency department for an episode of right faciobrachial hemisyndrome and speech disorder. The patient showed stable hemodynamic parameters, sinus rhythm on electrocardiogram, and a National Institutes of Health Stroke Scale (NIHSS) score of 6. Computed tomography (CT) of the brain excluded cerebral bleeding, whereas angiography reported an acute occlusion of the left M1 cerebral artery. Successful thrombolysis and mechanical thrombectomy were performed.

**Take-Home Messages**

• It is important to send all the specimens to the pathology for an early diagnosis.
• Each step for the correct diagnosis needs to be explained by clinical contest and physiopathological findings.

## Past Medical History

The young patient denied any previous disease. No history of drug assumption or abortions was reported. Smoking habit was present.

## Differential Diagnosis

The ischemic etiology of the brain lesion could be caused by several different diseases such as atheroma, paradoxical embolism, arrhythmias, antiphospholipid antibody syndrome, large vessel vasculitis or dissection, and neoplasia.

## Investigations

After fibrinolysis and mechanical thrombectomy, the patient had optimal neurological recovery, with the NIHSS score decreased to 0, and was admitted to the stroke unit. She underwent thrombophilia and immunological studies (protein S; resistance activated protein C resistance; lupus anticoagulant; antiphospholipid antibodies; antithrombin III; antinuclear antibody; extractable nuclear antigen; homocysteine; genetics factor II, V, and methylenetetrahydrofolate reductase; and tumor markers); all the study results were negative.

Transesophageal echocardiography was performed to exclude patent foramen ovale or cardiac thrombi. A small floating image in the aortic arch (3 mm) was detected and confirmed on the CT scans. No heteroplastic lesion was detected on the total body CT. It was assumed that the lesion was a complicated atheroma, and thus medical therapy was started and close follow-up was planned.

## Management

**Figure 1:**
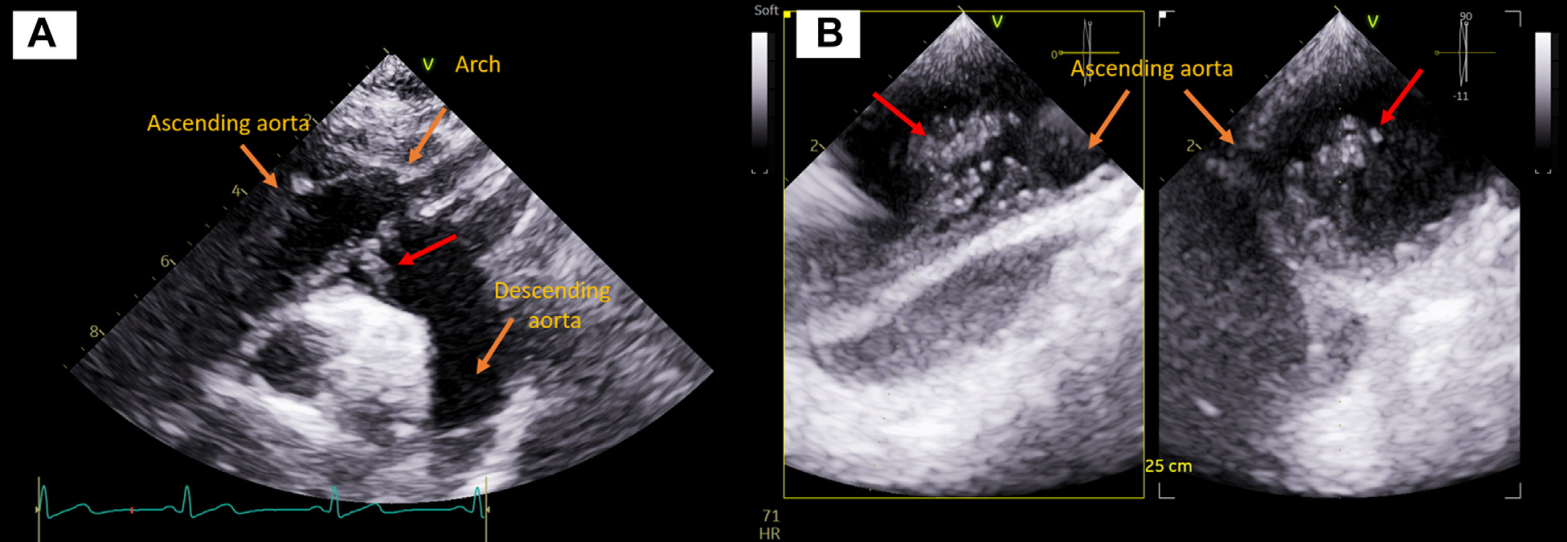
TTE and TOE Images (A) Transthoracic echocardiography (TTE) image shows the principal structures such as the arch, ascending aorta, and descending aorta indicated by orange arrows, while the lesion with irregular margins and distal appendages is indicated by the red arrow. (B) Transesophageal echocardiography (TOE) image shows the lesion at 25 cm from the buccal rim in the ascending aorta, indicated by the red arrow.

**Figure 2:**
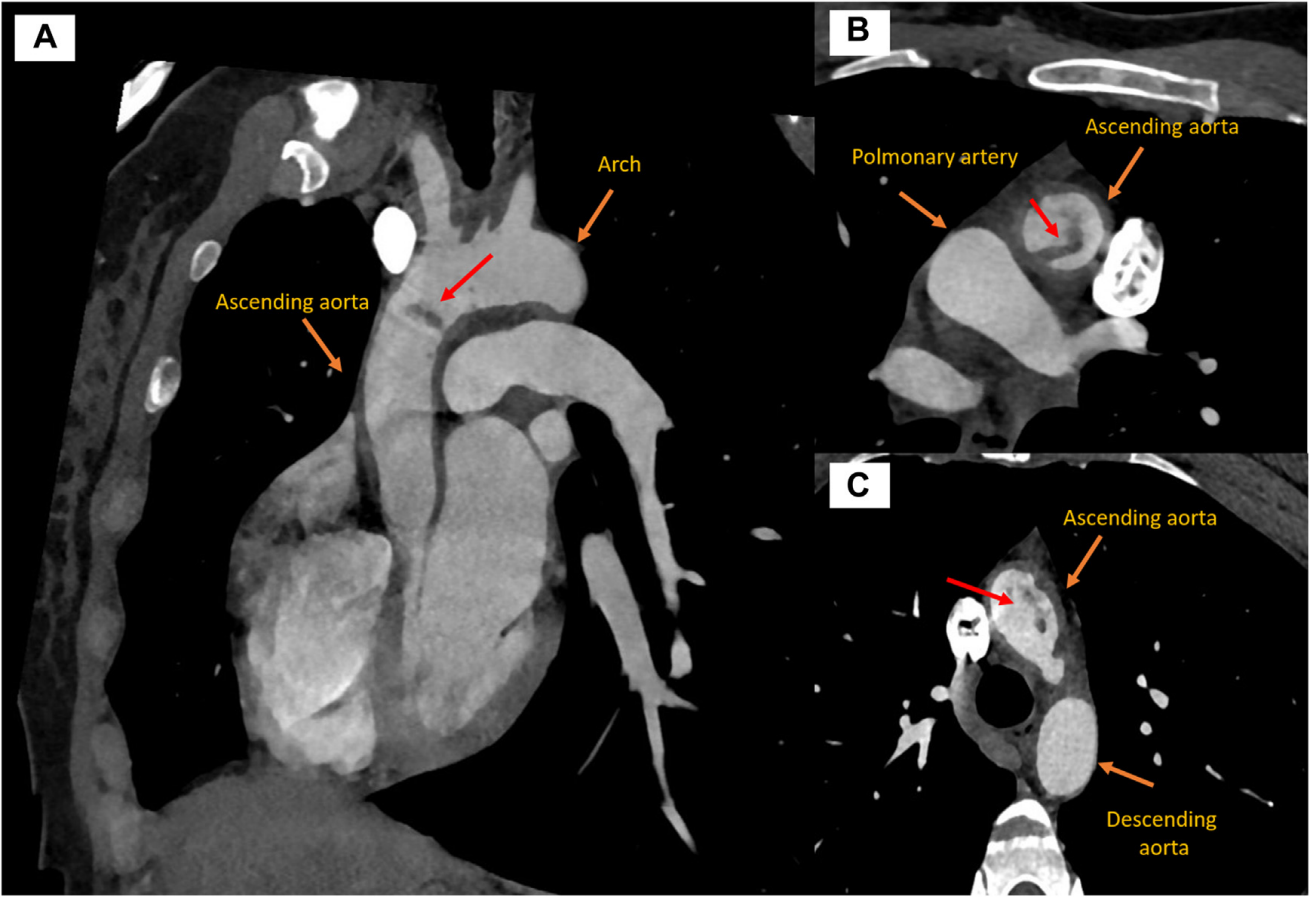
Computed Tomography Images (A to C) Images show the arch, ascending aorta, and descending aorta indicated by orange arrows, while the lesion in the ascending aorta is indicated by a red arrow.

Unfractionated heparin continuous infusion was contraindicated because of the recent cerebral event, and after 1 week of vitamin K antagonists bridging with low-molecular-weight heparin, the patient refused to undergo frequent blood sampling to monitor the international normalized ratio; thus, she was discharged with acetylsalicylic acid 100 mg (Cardioaspirin) daily and rivaroxaban 20 mg daily. The loading dose of rivaroxaban was not necessary because of the preliminary use of vitamin K antagonists. The 30-day course was uneventful, and she came back to the hospital for elective transthoracic echocardiography to check the evolution of the aortic wall lesion. The follow-up echocardiogram showed images of plus with irregular margins and distal appendages adherent to the aortic wall of the ascending aorta, which could, according to the first hypothesis, be referred to thrombi (Figure 1); CT scans confirmed the previous findings (Figure 2).

**Figure 3:**
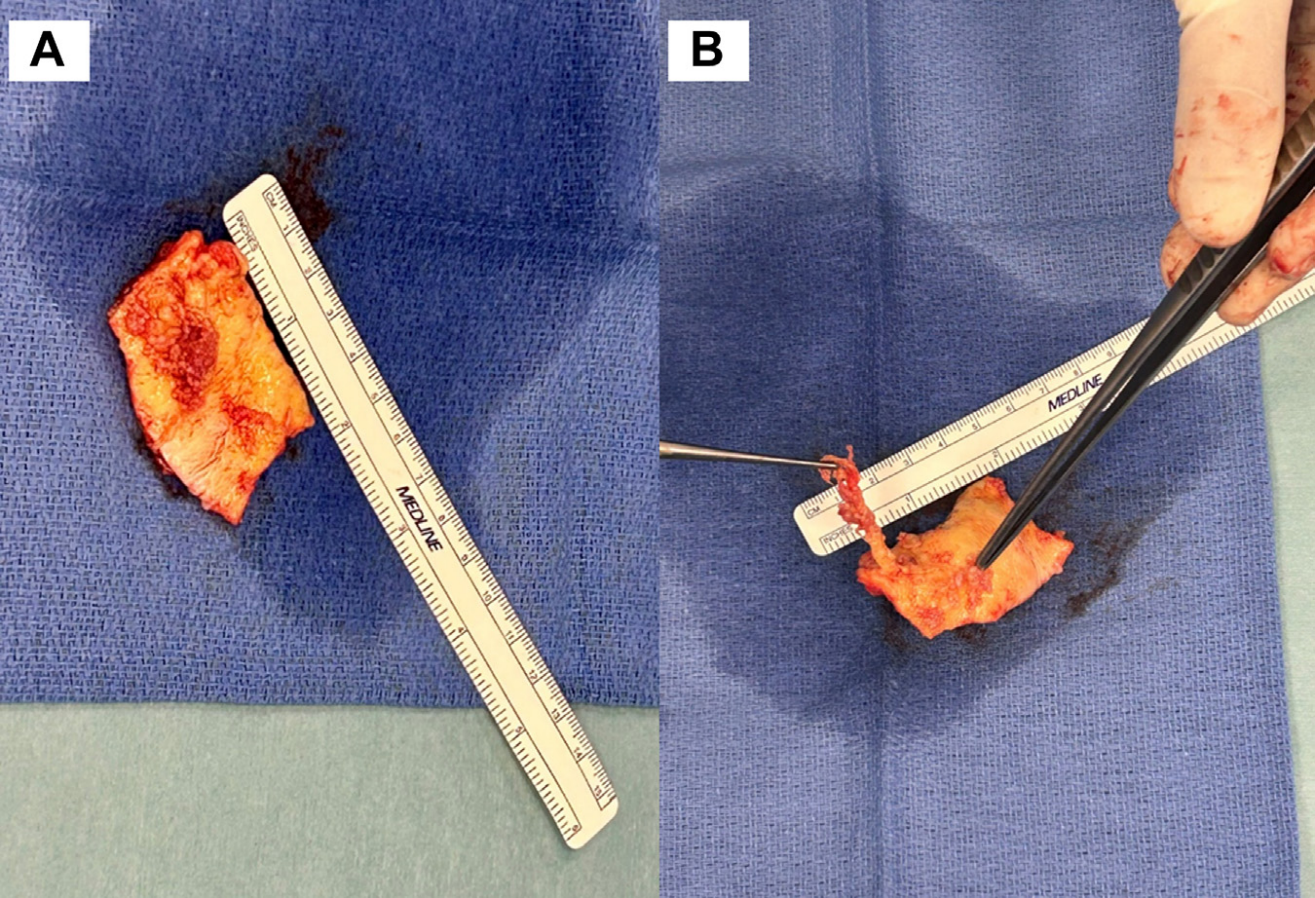
Surgical Findings (A and B) Macroscopically, the histological piece, measuring 35 × 30 × 7 mm, appeared internally with an irregular surface, and there were some grayish fragments adhering to the wall.

New echo assessment at 24 hours showed a slight progression of disease with mobile fragments just in front of the origin of the carotid artery. After heart team discussion, considering the high risk of thromboembolism and peripheral occlusion of other vessels, urgent cardiac surgery was indicated. The patient underwent successful surgical replacement of the ascending aorta and proximal aortic arch with a 26-mm dacron prosthesis. Tissue fragments inside the lumen of the bovine trunk were removed during circulatory arrest. Macroscopically, the histological specimen, measuring 35 × 30 × 7 mm, appeared internally with an irregular surface, and there were some grayish fragments measuring 14 × 1 mm adhering to the wall (Figure 3).

## Outcome and Follow-Up

**Figure 4:**
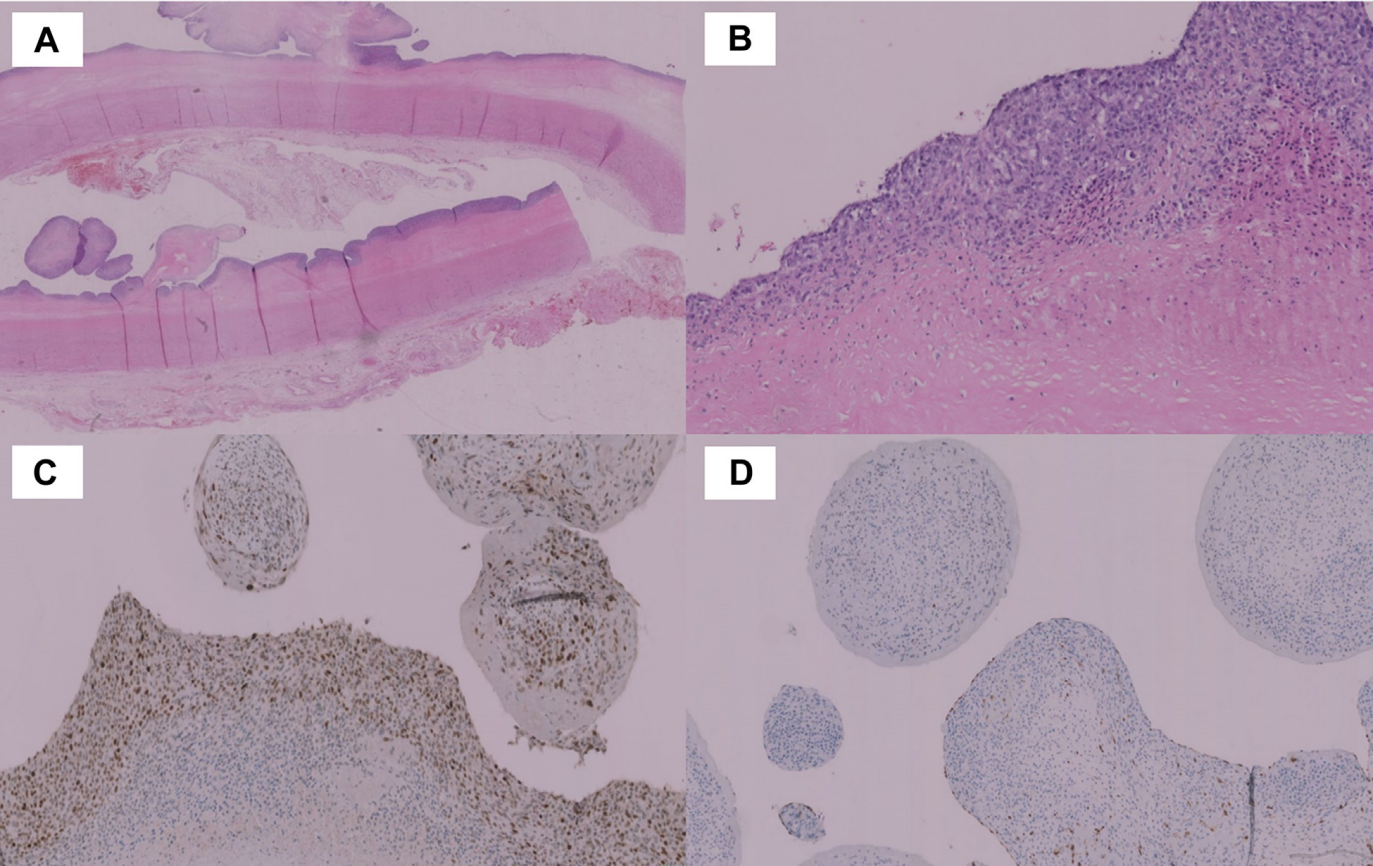
Histological Findings (A) The tumor shows a superficial spreading along the subendothelial portion of the aortic wall, forming polypoid masses protruding in the lumen, mimicking thrombi (hematoxylin and eosin stain, original magnification 2.5×). (B) At high magnification, tumor cells present a spindle to epithelioid configuration with aggressive changes, such as major nuclear pleomorphism, high cellular density, and abundant mitosis (hematoxylin and eosin stain, original magnification 20×). (C) There is immunohistochemical nuclear expression of anti-MDM2 antibody and negativity of endothelial markers such as ERG (D). ERG = erythroblast transformation-specific related gene; MDM2 = murine double minute 2.

Immunohistochemical investigation showed a malignant mesenchymal neoplasia compatible with intimal sarcoma. Proliferation was present on resection margins (Figure 4). At 1-month follow-up, the patient presented in good clinical condition with no cardiac or neurological symptoms (NIHSS score 0). Positron emission tomography CT showed early systemic neoplasia localizations, and the patient started medical therapy and oncological management in a cancer center specializing in rare pathologies outside the region.

## Discussion

The World Health Organization’s International Agency for Research on Cancer defines *intimal sarcoma* as a malignant mesenchymal tumor arising in the tunica intima of large arterial blood vessels of the systemic and pulmonary circulation. Intimal sarcomas of the aorta arise from subendothelial pluripotential cells within the intimal lining of the vessel wall. The mean age of diagnosis is variably reported, ranging in the fifth, sixth, and seventh decades of life, and the prognosis is poor. The median survival in patients without metastatic disease at the time of diagnosis is 20 months, and it is only 6 months when metastases are present. Clinical presentation is variably determined by tumor location, degree of vascular obstruction, embolic phenomena, and presence of metastatic disease. In many instances, aortic intimal sarcomas are often misdiagnosed as protuberant atherosclerotic disease or intimal thrombus and approximately 45% to 50% of patients with aortic sarcomas have metastatic disease at the time of diagnosis. Immunohistochemical testing is necessary for the diagnosis. Imaging involves ultrasound, echocardiography, CT, magnetic resonance imaging, and positron emission tomography, but the results of these radiological tests are often nonspecific. In our case, despite the use of transthoracic echocardiography, transesophageal echocardiography, and CT, this tumor was mistaken for a thrombus because of complicated atheroma. The CT scans did not show atheroma calcification, but this was due to the patient’s young age; this factor added confusion for the real diagnosis. Furthermore, the lesion was localized to a single aortic region, another feature uncommon in atherosclerosis. This case should underline the precious value of an early diagnosis through histological findings. We could have sent the embolectomy specimen to pathology for an early diagnosis, and the aortic tissue could also have been analyzed with an extemporaneous histological investigation to ensure efficient resection. Furthermore, even if the imaging tests are often nonspecific for the aortic sarcoma, there were subtle clinical suspicions that the lesion was not a common atheroma.

## Conclusions

Intimal sarcoma of the aorta is a subtype of a primary malignant tumor of the aorta—very rare and highly aggressive. Symptoms are often nonspecific, and the clinical presentation is variably determined by tumor location. Treatment is challenging in many cases, and the prognosis is poor; therefore, a multidisciplinary approach is mandatory in all cases. For each step, different specialists were involved. Neurologists suspected that the stroke had a cardiovascular origin; cardiologists and radiologists contributed to the clinical and imaging findings; cardiac surgeons removed the lesion; and pathologists and oncologists had a role in the final diagnosis and therapy, respectively.

## Funding Support and Author Disclosures

The authors have reported that they have no relationships relevant to the contents of this paper to disclose.

